# Leafcutter Bee Nests and Pupae from the Rancho La Brea Tar Pits of Southern California: Implications for Understanding the Paleoenvironment of the Late Pleistocene

**DOI:** 10.1371/journal.pone.0094724

**Published:** 2014-04-09

**Authors:** Anna R. Holden, Jonathan B. Koch, Terry Griswold, Diane M. Erwin, Justin Hall

**Affiliations:** 1 Entomology Section, Natural History Museum of Los Angeles County, Los Angeles, California, United States of America; 2 Department of Biology and Ecology Center, Utah State University, Logan, Utah, United States of America; 3 USDA-ARS Pollinating Insect Research Unit, Utah State University, Logan, Utah, United States of America; 4 Museum of Paleontology, University of California, Berkeley, Berkeley, California, United States of America; 5 Dinosaur Institute, Natural History Museum of Los Angeles County, Los Angeles, California, United States of America; University of Kansas, United States of America

## Abstract

The Rancho La Brea Tar Pits is the world’s richest and most important Late Pleistocene fossil locality and best renowned for numerous fossil mammals and birds excavated over the past century. Less researched are insects, even though these specimens frequently serve as the most valuable paleoenvironemental indicators due to their narrow climate restrictions and life cycles. Our goal was to examine fossil material that included insect-plant associations, and thus an even higher potential for significant paleoenviromental data. Micro-CT scans of two exceptionally preserved leafcutter bee nest cells from the Rancho La Brea Tar Pits in Los Angeles, California reveal intact pupae dated between ∼23,000–40,000 radiocarbon years BP. Here identified as best matched to *Megachile* (*Litomegachile*) *gentilis* Cresson (Hymenoptera: Megachilidae) based on environmental niche models as well as morphometrics, the nest cells (LACMRLP 388E) document rare preservation and life-stage. The result of complex plant-insect interactions, they offer new insights into the environment of the Late Pleistocene in southern California. The remarkable preservation of the nest cells suggests they were assembled and nested in the ground where they were excavated. The four different types of dicotyledonous leaves used to construct the cells were likely collected in close proximity to the nest and infer a wooded or riparian habitat with sufficient pollen sources for larval provisions. LACMRLP 388E is the first record of fossil *Megachile* Latreille cells with pupae. Consequently, it provides a pre-modern age location for a Nearctic group, whose phylogenetic relationships and biogeographic history remain poorly understood. *Megachile gentilis* appears to respond to climate change as it has expanded its distribution across elevation gradients over time as estimated by habitat suitability comparisons between low and high elevations; it currently inhabits mesic habitats which occurred at a lower elevation during the Last Glacial Maximum ∼21,000 years ago. Nevertheless, the broad ecological niche of *M. gentilis* appears to have remained stable.

## Introduction

*Megachile* Latreille [1] is a large, worldwide genus of approximately 1,500 species of largely leafcutting, solitary bees. In the Western Hemisphere they inhabit temperate, arid, and tropical regions extending from Alaska to Tierra del Fuego [2]. There are 118 species native to North America [2]. The abundance of megachilids in California is not surprising given the wide diversity of habitats and microclimates [3], [4].

Leafcutter bees are named for their use of leaf pieces in nest building. They constitute *Megachile* belonging to Michener’s Group 1 [5] in which bees frequently construct two or more cells in a linear series. Their nesting sites are found under the bark of dead trees, in stems, in the burrows of wood-boring insects or in burrows self-dug in loose soil or those made by other animals [2], [5], [6]. The females use their sharp, serrated, scissor-like mandibles to cut oblong and circular leaf pieces, most likely from plant sources near the nest [2], [5]. They line the nest cavities with overlapping layers of the oblong-shaped leaf disks. The leaf edges are compressed to extrude sap that, in combination with saliva, creates a glue-like substance that keeps the cells sealed and intact [2]. Each cell is provisioned with pollen and nectar by the female before she deposits a single egg on the food mass. After depositing the egg, she seals the cell with one to several circular leaf “caps” [2], [5], [6]. After a few weeks, depending upon species, the eggs hatch, and the larvae develop through multiple instars and feed on the provisions. Mature larvae spin cocoons of two or more layers of silk and diapause as prepupae. Cocoons are sturdy structures [7] made increasingly airtight by the larva’s secretion of a brown liquid that fills and hardens the interstices of the silk layers [6]. This application binds the silk mesh and makes the cocoon extremely durable. Simultaneously, this fastens the cocoon’s outer surface to the surrounding leaf disks that firmly hold the structure together. The larvae subsequently pupate and emerge as adults by chewing their way out through the cap.

That females may spend the majority of their time collecting pollen and nectar to provision their young [2] and construct intricate nests with specific materials indicates a very complex and highly evolved plant-insect interaction, and strongly suggests a long evolutionary history [8]. The use of leaf disks of various sizes, shapes, and textures also reflects highly complex and evolved behavior [2], [8], [9], [10].

As currently known, the megachilid fossil record is restricted to the Cenozoic based on body fossils preserved as compressions and three-dimensionally preserved in amber, as well as trace evidence from fossil angiosperm leaves whose margins show smooth-edged oblong and circular cutouts [8], [11], [12]. Engel [13]–[15] and Engel and Perkovsky [11] have compiled the evolutionary history and an overview of the body fossil record, respectively. Morphological data (body fossils and leaf cutouts attributed to *Megachile*) and molecular data do not always agree on the time divergence of the genus [8], [11]–[14], [16]–[22]. Although the phylogenetic relationships and evolutionary history of the genus have become clarified as more studies incorporate molecular data [23], the fossil record remains incomplete and some specimens assigned to Megachilidae may need revision. For example, molecular data suggest that Megachilidae arose in the Cretaceous about 140–100 mya, but the genus *Megachile* is estimated to have originated only 22 mya [23]. However, leafcutters are derived species of *Megachile* and therefore, the fossil record based on leaf cutouts from the Early to middle Eocene in North American and Europe [16]–[20] suggests that basal divergences in the Megachilini occurred earlier in the Paleocene or Latest Cretaceous [11].

Here we report on fossil *Megachile* nest cells with pupae (LACMRLP 388E) recovered from the Late Pleistocene Rancho La Brea Tar Pits in southern California. Though geologically young, this is the first report of three-dimensionally preserved *Megachile* nest cells that shows rare preservation and life-stages. The pupal morphology, nesting behavior, and cell construction of LACMRLP 388E best match *M. gentilis,* a member of the native Nearctic *Litomegachile* Mitchell [24], a subgenus that today ranges from southern Canada to Cuba and southern Mexico [5], [25], [26].

The discovery of LACMRLP 388E provides valuable information for better understanding the environmental conditions of southern California in the Late Pleistocene. By setting specimens within a geological as well as an ecological context, Quaternary fossils are shown to be valuable precursors to modern biota [27]. Although the asphaltic deposits at Rancho La Brea are most often associated with vertebrate remains from saber-toothed cats and mammoths, the insects and plants found there are also significant fossils because they are original material, and thus, intact, three-dimensional, and structurally complex. As such they can provide the most valuable paleoenvironmental information for the richest Ice Age fossil locality. Our goal was to synchronize data by identifying both nest cell insect and plant material in order to make the significant paleoenvironmental inferences possible. These efforts resulted in environmental data, that a mesic environment occurred at a lower elevation than today at Rancho La Brea, the well-established provenance for LACMRLP 388E. This research also resulted in new information on *M. gentilis*, including diagnostic features of its nest cell architecture and insight regarding its relatively conserved ecological niche since the Late Pleistocene.

In additional to its role as a sensitive paleoenvironemental indicator and providing new information on *M. gentilis*, LACMRLP 388E is of rare value because its remarkable preservation, especially of the intact pupae, is of a standard unusual even for fossil material from Rancho La Brea.

## Results

### LACMRLP 388E

#### Provenance and age

**Figure 1:**
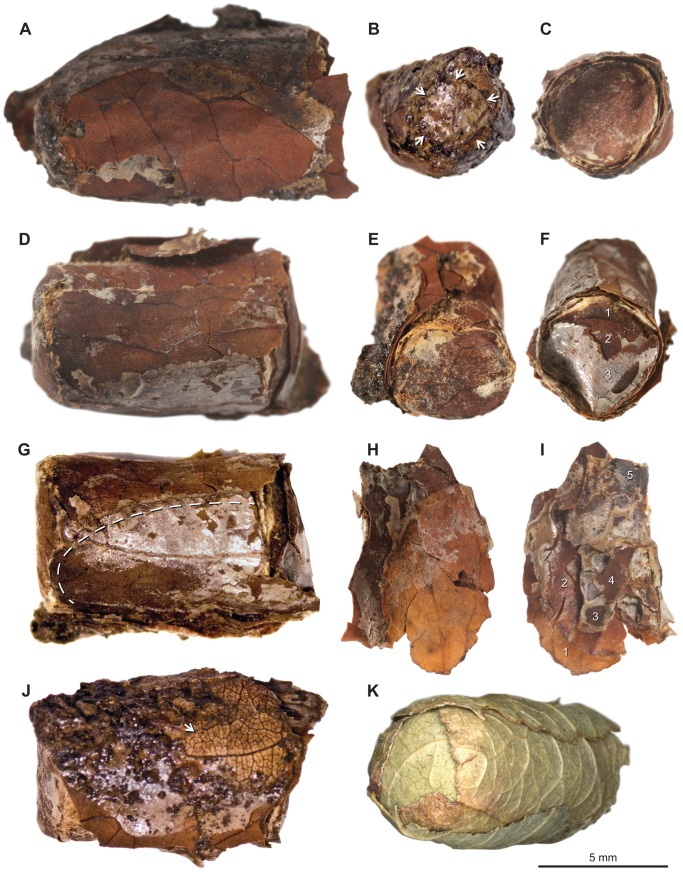
Images showing nest cell construction and modern nest cell of *Megachile gentilis* for comparison. A–C = LACMRLP 388Ea; D–G, J = LACMRLP 388Eb; H, I = LACMRLP 388Ec. (**A**) Nest cell containing male pupa showing cylindrical shape, tapered, rounded bottom at left typical of the first constructed cell, and remains of oblong leaf disc with Type 1 venation. (**B**) Bottom of first constructed cell (containing male pupa) with possible portion of bottom circular leaf disk visible and outlined with arrows. (**C**) Cap of nest cell containing male pupa. (**D**) Nest cell containing female pupa. Arrow shows margin of oblong leaf disc with Type 2 venation. (**E**) Circular, bottom disc of nest cell (containing female pupa). In life, this end abutted the anterior end of LACMRLP 388Ea. (**F**) Cap of nest cell containing female pupa. (**G**) Nest cell of female pupa showing oblong side wall leaf cutout which does not reach bottom of cell and is instead supported by circular bottom disc. (**H**) Remains of oblong leaf disc with relatively smooth-cut margins and Type 3 venation. (**I**) View showing five overlapping oblong disks (1–5) comprising the sidewalls and circular bottom disk. discs. (**J**) Nest cell containing female pupa showing Type 4 venation on upper, right corner. (**K**) Nest cell of modern *M. gentilis*, showing circular disc bottom and oblong, sidewall leaf.

LACMRLP 388E was excavated in 1970 from the northeast corner of Pit 91, one of approximately 100 asphaltic deposits at Rancho La Brea in Los Angeles. It was found as one intact specimen (LACMRLP 388E) in an area with asphalt-impregnated sediment and bone at a depth of 205 cm. However, on subsequent handling the two nest cells separated (LACMRLP 388Ea, Fig. 1*A–C* *;* LACMRLP 388Eb, Fig. 1*D–G, J*) along with a portion of the outer leaf layers (LACMRLP 388Ec) (Fig. 1*H, I*).

Fifty fossil bones from Pit 91 have been dated between 23,000–40,000 years BP based on radiocarbon dated bone collagen [28]. This is with the exception of one, anomalous bone on the opposite side of the pit from LACMRLP 388E, which was dated to 14,000 years BP [28]. Furthermore, LACMRLP 388E was recovered from a depth of less than 243 centimeters that included a cluster of bones dated to ages older than 35,000 years BP, increasing the likelihood that LACMRLP 388E is between 35,000 and 40,000 radiocarbon years BP [28]. That the nest cells were constructed underground but close to the surface before becoming embalmed in asphalt-rich matrix indicates that they may have been near an asphalt pipe from which oil soaked into the surrounding sediment [28].

Many aspects of LACMRLP 388E nest cell construction increased the probability of fossilization in asphalt. An underground burrow provided a protected environment from the elements, as well as many predators. Glue-like saliva containing anti-bacterial and anti-fungal properties likely prevented decay before fossilization [5], [29], [30]. Water-resistance was created from sap extruded from the cell leaf pieces, tight assembly of multiple leaf layers, the hydroscopic properties of the leaf epidermis, and the internal silk cocoon which was hardened and airtight from larval secretions plus lipids from the pollen provisions. Finally, the life stage–a bee in the pupal stage–would be less vulnerable to desiccation than a larva whose exoskeleton is less strongly sclerotized.

#### Pupae

**Figure 2:**
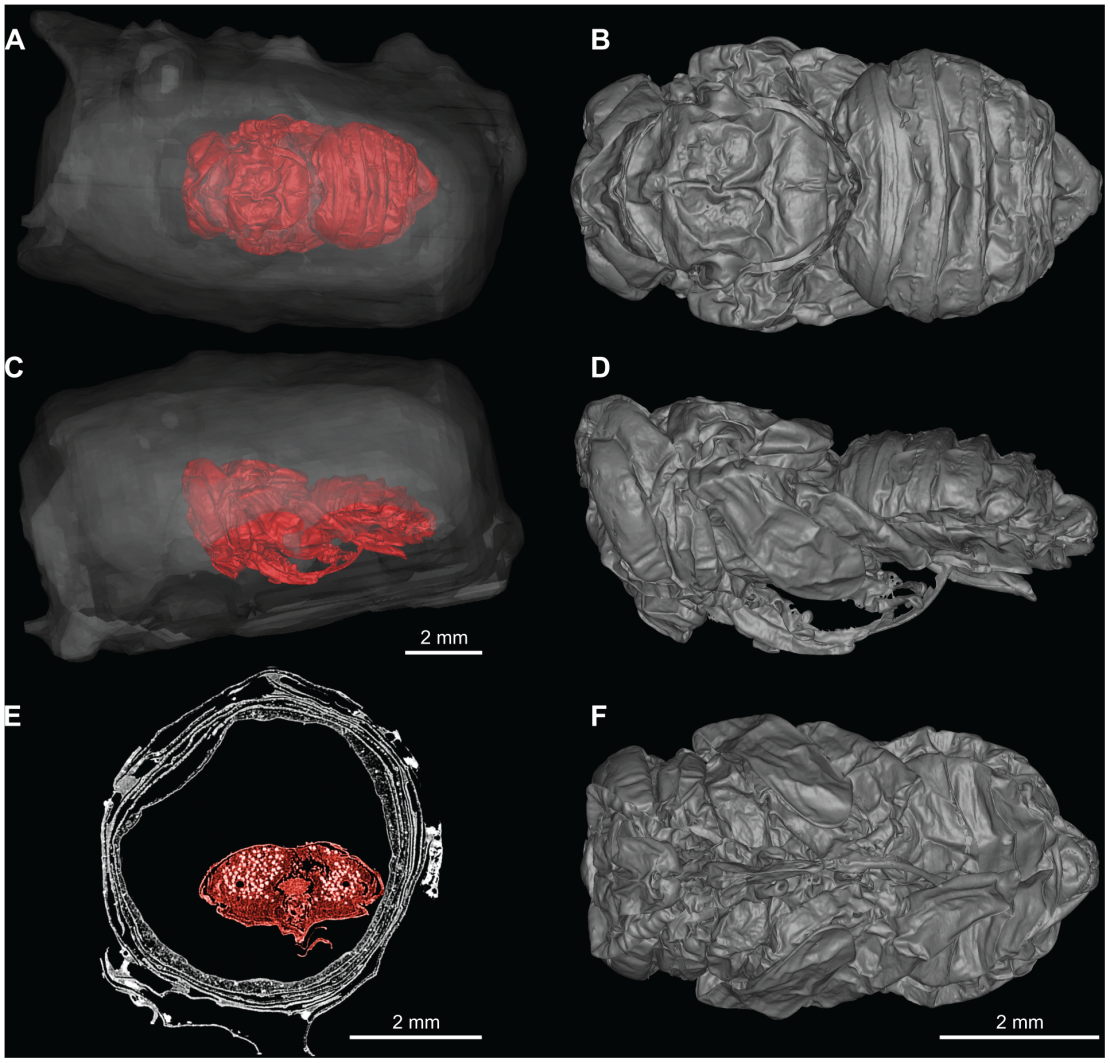
Micro-CT scans of LACMRLP 388Ea showing male pupa and its position within the nest cell. (A) Dorsal view of pupa within nest. (B) Dorsal view of pupa. (C) Lateral view of pupa within nest. (D) Lateral view of pupa. (E) Cross-section of nest and pupae. (F) Ventral view of pupa.

**Figure 3:**
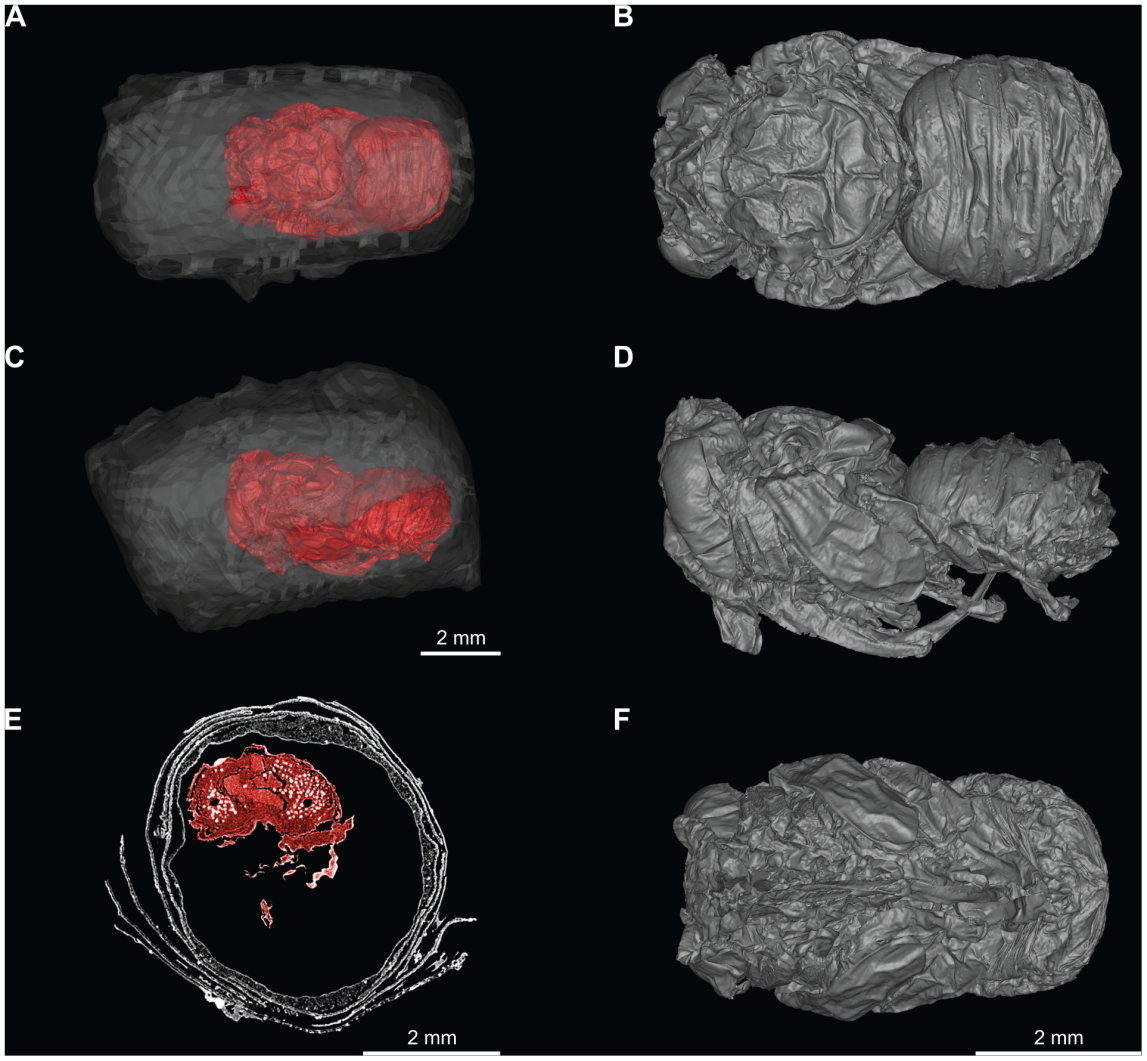
Micro-CT scans of LACMRLP 388Eb showing female pupa and its position within the nest cell. (A) Dorsal view of pupa within nest. (B) Dorsal view of pupa. (C) Lateral view of pupa within nest. (D) Lateral view of pupa. (E) Cross-section of nest and pupae. (F) Ventral view of pupa.

**Figure 4:**
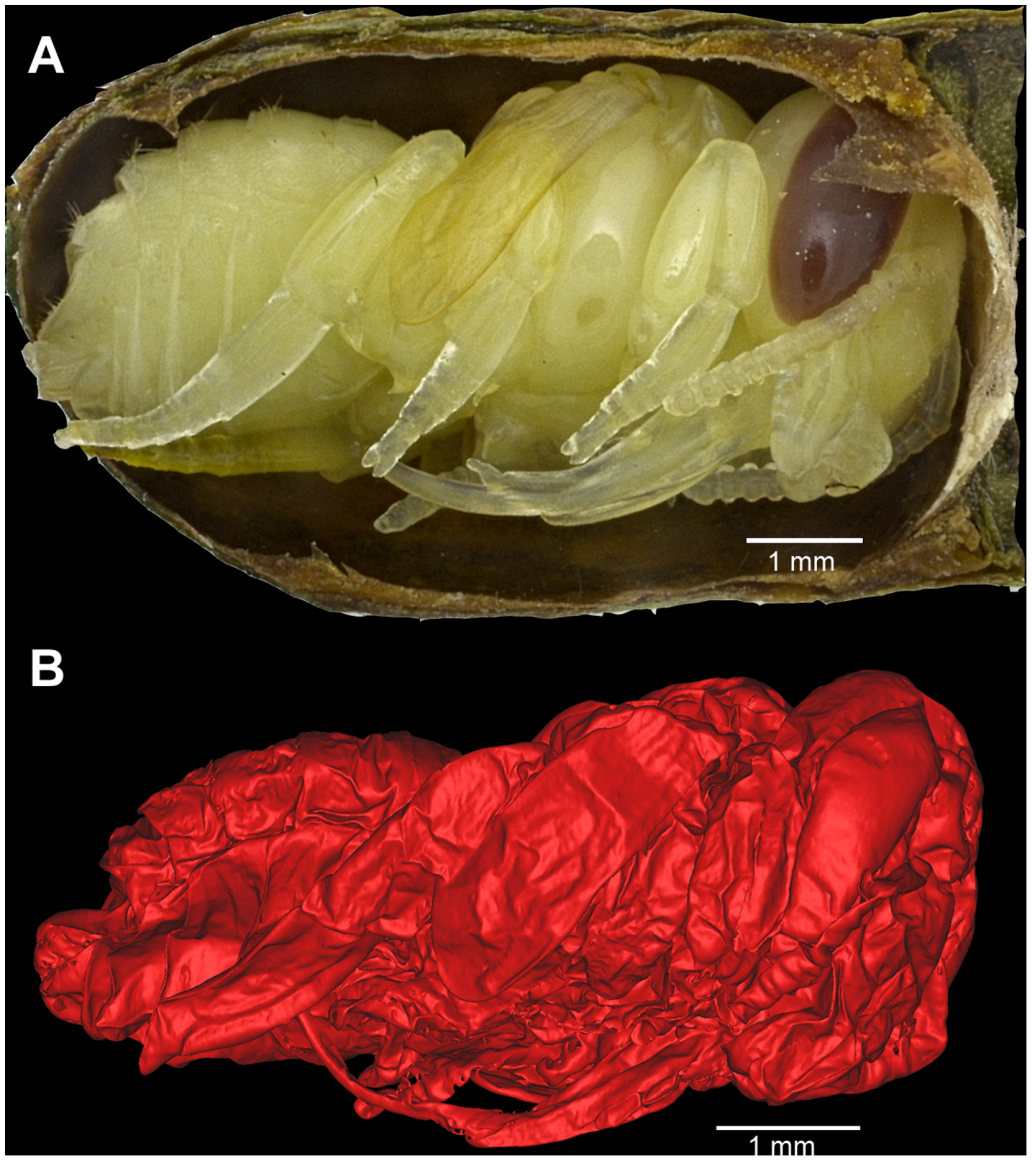
Comparison between (A) a female modern leafcutter bee (*Megachile rotundata*) and (B) LACMRLP 388E male and in order to display obvious similarity in general morphology at pupal stage.

Originally identified on-site as a bud (Obermayr, Pit 91 field notes p. 1770, 1970), LACMRLP 388E was later tentatively identified to the bee family Apidae. Micro-CT scans of LACMRLP 388E reveal a pupa within each cell, indicating that the specimen was preserved sometime during flowering season (Fig. 2*A, C* and Fig. 3*A, C* *,* Videos S1-6) since *Megachile* do not overwinter as pupae. A comparison of LACMRLP 388E to a modern leafcutter bee (*Megachile rotundata*) show obvious similarity in general morphology at pupal stage (Fig. 4). While nest cells are designed to house *Megachile* offspring, the megachilid cleptoparasite *Coelioxys* may usurp nest cells. Images of the pupae in dorsal and ventral views (Fig. 2*A, B, F* and Fig. 3*A, B, F*, Videos S3, 4) have the same general body morphology, presence of setal pits on scutum, the long labrum, and oval shape of the metasoma of *Megachile*, confirming the identity of the nests based on cell construction and structure. The shape of the metasoma and posteriorly rounded mesosoma eliminates the possibility of *Coelioxys*, which would have a more elongate, tapered metasoma in dorsal and ventral views and pointed axillae. One pupa is present in each cell and both sexes are represented (Fig. 2 and Fig. 3, Videos S1-6), the male being distinguished by a dorsal horizontal ridge on metasomal segment six (Fig. 2. *A, B*). Because the cells were connected *in situ*, and are not cleptoparasitized, they can only be the same species.

### Nest Cell Construction

#### Nest cell morphology

The two nest cells that constitute LACMRLP 388E are approximately 10.5 mm in length and 4.9 mm in diameter, constructed of oblong and circular leaf cutouts (Fig. 1*A–J*). The nest cells when found were joined end to end, the male cell in Figure 1*A* being the cell first assembled in the ground, while the female cell (Fig. 1*D, G, J*) would have been closer to the nest entrance. They were held together by additional cutouts that lined the nest cells but some dislodged after excavation and were lost. With subsequent handling another small portion sloughed off but was conserved. It consists of an additional five oblong-shaped disks (Fig. 1*H, I*). Outward morphology (Fig. 1*A–G, J*) and cross-sectional view of the cells (Fig. 2*E* and Fig. 3*E*, Video S7) show the sides and inner lining consist of 5–7 overlapping smooth-edged, oblong-shaped disks, the ends capped by small circular disks (Fig. 1*C, F*, Video S7). At least three discs cap the end of the male cell (Fig. 1*F*).

Most often, oblong leaves form the side walls and are bent into a cup at the proximal end (furthest from the nest entrance) that is glued with saliva and extruded leaf sap. A cap consisting of a series of layered circular discs is placed on the distal end (closest to the nest entrance). However, the separation of the two cells reveals the nest cell containing the female pupa includes a circular cap (Fig. 1*E*), as well as a circular bottom (Fig. 1*F*). Certain *Megachile* species insert a circular disc to provide an internal brace for the nest cell if the cup pieces are looser and less glued together than as constructed in other species [9], [10], [31]. Therefore, we presume that the nest cell containing the male pupa was the first cell in the cavity because it is hard to distinguish if the circular base is formed from the folded edges of the larger leaf fragments or the insertion of a smaller internal disc (Fig. 1*A, B* arrows). Literature on *Megachile* nest cell construction informs that the inclusion of a circular base is an uncommon construction for the genus [*2]*, *[5*].

Among extant Nearctic *Megachile* the small size of the cells and the use of leaves in nest construction limit candidate species to small members of the subgenus *Litomegachile*, specifically *M. brevis* Say, *M. gentilis* (Fig. 1*K*), *M. onobrychidis* Cockerell and *M. pseudobrevis* Mitchell. *Megachile brevis* and *M. pseudobrevis* are excluded from consideration because the former uses petals in cell construction [32] and the latter’s current distribution is restricted to a limited range in the southeastern United States [25]. Exclusion of *M. pseudobrevis* is further supported by the incorporation of petals in two nests reported from Florida [33]. Further examination of the distinct nest cell architecture and exclusive use of leaves as cell material for LACMRLP 388E (Video S7), in combination with pupal morphology (Videos S1-6), indicate that these cells belong to *M. gentilis*, a species whose current distribution is concentrated in the southern portion of the Pacific coast, the Southwest, northern Mexico, and Texas [25, Discover Life *Megachile gentilis*, http://www.discoverlife.org/mp/20m?kind=Megachile+gentilis, 2013] rather than *M. onobrychidis* (see below).

#### Plant material used

Since *Megachile* typically cut segments from the leaf margin, we can identify at least four, possibly five different taxa used in construction of the two cells based on the different patterns of venation (Figs. 1*A, D, G, J*). Dicotyledonous leaves typically have one or more large primary veins, with a reticulate network of successively narrower second, third, fourth, and fifth order veins in a pattern consistent within a species [34]. Although leaf venation is especially useful in fossil plant identification, venation characters are most reliable in combination with other morphological details such as leaf shape and size, shape of the leaf apex and base, and margin and petiole features [34], characters unavailable in the small areas of the leaf cutouts. Regrettably, this makes it difficult to identify with confidence the fragments to a specific species or even family. However, leaf texture, thickness, and the venation that is present are consistent with the cutouts being from woody trees, shrubs, or vines rather than herbaceous taxa, the leaves of which do not preserve well as fossils. Some *Megachile* include petals in their cells but venation of the cutouts of LACMRLP 388E is not consistent with petal venation [*35]*–*[37*], nor is their thickness (Video S7).

### Historic and Contemporary Environmental Niche Space

**Figure 5:**
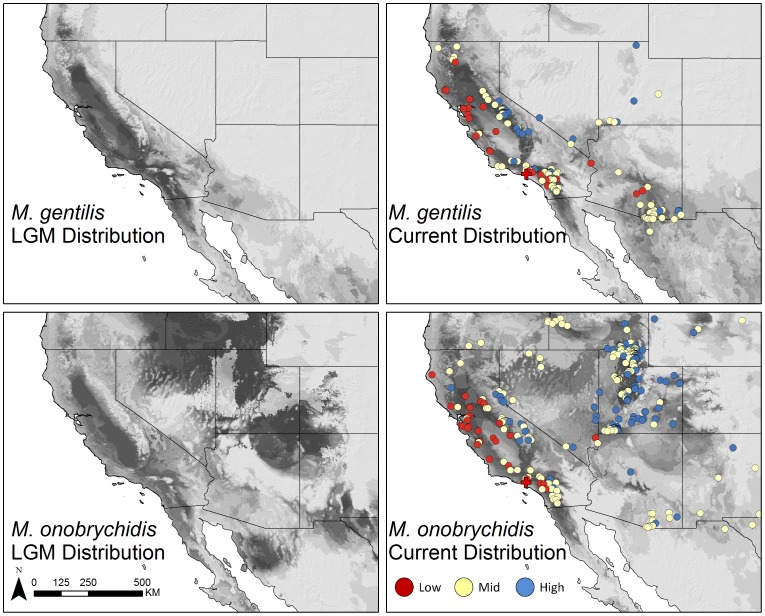
Estimated geographic distribution of *M. gentilis* and *M. onobrychidis* at present and during the last glacial maximum (LGM;∼21 000 years before present) in the western United States. Darker colors represent high MaxEnt habitat suitability (values closer to H = 1), whereas lighter colors represent low HS (values closer to HS = 0). Black squares represent georeferenced point localities of the respective species (Table S1). Red crosshairs represent the location of the La Brea Tar Pits in Los Angeles, California. Habitat suitability distributions of both species are based on eight bioclimatic variables.

**Figure 6:**
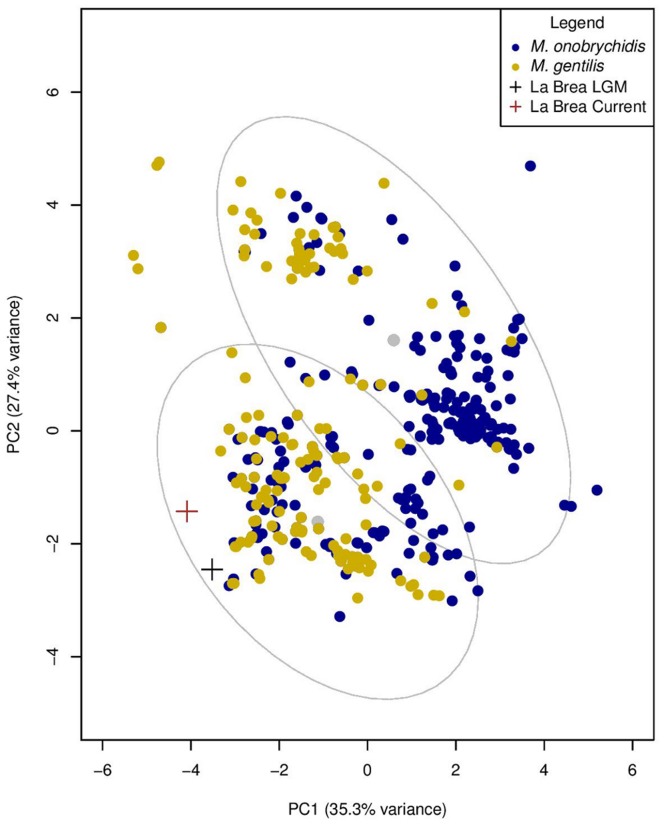
Principal components analysis of environmental space use by *M. gentilis* and *M. onobrychidis*. Estimates of environmental space use include eight bioclimatic variables, as well as, Cartesian (*i.e.,* latitude and longitude) and elevation variables. Clusters reflect 99% data concentration ellipses determined with the *scatterplot* (*car* library) functions in R v3.3.0 (R Core Team 2012).

We further researched the best species match for the La Brea bees by examining the historic environmental niche space of the La Brea *Litomegachile* relative to the contemporary environmental niche of *M. gentilis* and *M. onobrychidis,* the species whose nest cell morphology, distribution, size, and nesting behavior is closest to *M. gentilis*. To estimate the contemporary environmental niche space of *M. gentilis* and *M. onobrychidis* we associated a suite of informative bioclimatic variables with georeferenced natural history records (see Materials and Methods). We then constructed the historic environmental niche space of the La Brea bees by associating bioclimatic variables values of the Last Glacial Maximum (LGM, ∼21,000 years ago) with georeferenced coordinates of the Rancho La Brea Tar Pits (latitude = 34.063068, longitude = −118.355412). While both species are broadly sympatric throughout California at present (Fig. 5), we found that the historic environmental niche of the La Brea bees in the Los Angeles Basin during the LGM fell within the 95% data concentration ellipse of the contemporary environmental niche space for *M. gentilis* in a principal component (PC) analysis (PC 1 = 32.7%, PC 2 = 26.5%) (Fig. 6). Furthermore, LGM habitat suitability estimates based on contemporary habitat suitability models (HSM) suggest that *M. gentilis* had a higher probability of being distributed at low elevations in the Los Angeles Basin than *M. onobrychidis* (Fig. 5, Fig. S2) (see Material and Methods).

Due to broad overlap in geography and environmental niche space between *M. gentilis* and *M. onobrychidis*, we further tested the assignment of the historic environmental niche of the La Brea bees using cluster analysis. Ninety percent of the contemporary *M. gentilis* distribution records, along with the La Brea fossil distribution record and associated historic environmental space values, were assigned to the contemporary environmental niche space of *M. gentilis* (Fig. 6). Sixty-five percent of the contemporary *M. onobrychidis* distribution records were assigned to the contemporary environmental niche space of *M. onobrychidis* (Fig. 6). The high assignment of the contemporary *M. gentilis* records to the *M. gentilis* environmental niche space is likely due to the narrow environmental distribution of the species. In comparison to *M. onobrychidis*, *M. gentilis* is distributed across mesic environments, where temperatures rarely fall below freezing during the coldest months (Fig. S1), with ample precipitation during the wettest months (Fig. S1). The ability for *M. onobrychidis* to exist in highly variable environments may have allowed that species to endure prolonged, freezing temperatures during overwintering periods, and disperse across a broad range of environments, whereas the environmental specificity of *M. gentilis* supports its narrow distribution in the Pacific US (Fig. 5). Cluster and principal component analysis, as well as estimates of habitat suitability during the LGM are consistent with our species identification of the La Brea bees unearthed in the Rancho La Brea Tar Pits in southern California as *M. gentilis*.

## Discussion

The provenance of specimens recovered from Rancho La Brea cannot always be discerned because alluvial wash may have deposited some of the recovered material at a distance from their original location [38]. Nevertheless, the remarkable preservation and lack of water damage to LACMRLP 388E indicates with near certainty that it was assembled in the ground on the site of Pit 91 where it was found. The provenance of the specimen, in combination with environmental niche models and morphometric investigations, constitutes strong and comprehensive evidence to support confident fossil identifications and subsequent paleoenvironmental inferences for the Late Pleistocene, an epoch of particular climatic variation.

Because the leaves used in nest construction were collected in close proximity to each other and at the site of deposition, we can infer from their identification that a woody and riparian habitat existed at Rancho La Brea ∼23,000–40,000 radiocarbon years BP. This inference is supported by Maxent habitat suitability models which suggest that *M. gentilis* was distributed in a mesic environment that likely occurred at a lower elevation during the Last Glacial Maximum (LGM) ∼21,000 radiocarbon years BP (Fig. S1, Fig. S2). Presently, *M. gentilis* occurs in higher elevation habitats that constitute the perimeter of its projected habitat suitability during the LGM (Fig. 4). These areas surrounds the Los Angeles Basin in which Rancho La Brea is situated (Fig. 4).

Fossil plants from Pit 91 excavations have been grouped into four categories including a riparian association which includes plants from flood plain and canyon habitats [39]. Aquatic and moisture-loving plants indicate a permanent water source near Rancho La Brea [39], [40]. But additional plants from Pit 91 indicate other associations such as coastal sage scrub, chaparral, and deep canyon [39]–[41]. Most of the excavated plants probably inhabited Rancho La Brea, but others may have been transported by streams or floods [39], [41]. LACMRLP 388E suggests for the immediate Rancho La Brea area the presence of a gallery forest within a riparian zone. These forests exist along watercourses often in arid regions and standout in contrast to e.g. an adjoining grassland or open woodland habitat. The indication of a mesic environment at Rancho La Brea is relatively consistent with coastal run-off records [42] which indicate increased precipitation, possibly associated with increased glaciation [43], at ∼25,000–20,000 years ago. Relative comparison of the geographic distribution of habitat suitability between the contemporary and LGM models reveals that *M. gentilis* has remained virtually unique to California and southwestern Arizona (Fig. 5) where the average temperature of the coldest quarter rarely falls below freezing (0°C). The contemporary distribution of *M. gentilis* is limited to mesic environments where the difference between the maximum of the warmest month and the minimum of the coldest month is centered around 33°C (Temperature Annual Range, Fig. S1). That *M. gentilis* has retained its abiotic niche since the LGM provides a strong benchmark with which to compare the climate restrictions of other fossils from Rancho La Brea.

Most animals and plants excavated from Rancho La Brea are extant, and those that have become extinct are mostly mammals such as common, larger carnivores (e.g. saber-toothed cats and dire wolves), common, medium-to-large herbivores (e.g. the western horse, mastodonts and mammoths), and some birds [39]. Only two species of insects recovered from Rancho La Brea, scarabs that relied on dung from mammals that became extinct, may have died out as well [39], [44]. The current geographic distribution of most of the insect species from Rancho La Brea occurs in warmer parts of the United States and Mexico. Insect damage to the fossil bones attributed to tenebrionid and dermestid beetles is consistent with a warm period of at least 4–5 months at the time that the fossils were trapped [45]. This differs from interpretations of Late Pleistocene climatic conditions in southern California based on pollen from deep sea cores which indicate a shift to a cooler environment, as indicated by a transition from predominantly hardwood scrub-oak vegetation to coniferous pines and juniper [46]–[48] during the Last Glacial Interval from 24,000–14,000 years BP [46]. However, offshore palynological records may not reveal short-term increases in temperature that would result in both the colonization of insects with warm climate restrictions for brief periods due to their higher mobility, and in increased asphalt entrapment from more active seeps [45]. The current geographic distribution and climate restrictions of insect species preserved at Rancho La Brea, along with the physical properties of natural asphalt, indicate that the fossil insects represent warmer intervals of the Late Pleistocene [45]. But this does not resolve the discrepancy that some fossils recovered from Rancho La Brea such as *M. gentilis* inhabit mesic environments, while most of the tenebrionid beetles preserved at Rancho La Brea inhabit dry scrub and woodland habitats [49]. Further research on insects that can be dated to a specific time period, and that also have a clear provenance for Rancho La Brea, as well as narrow climate restrictions, will greatly enrich our understanding of the paleoenvironment, as well as climate variation during the Late Pleistocene in southern California.

Aside from providing paleoenvironmental data, LACMRLP 388E enhances the fossil record of megachilids and the genus *Megachile* by providing the first reported specimen from the Pleistocene. This contributes to a better understanding of a genus whose phylogenetic relationships are still being researched.

The examination of LACMRLP 388E also presents neontologists with important information. O’Toole and Raw [2] state that, despite the diversity of nest sites and habitats exploited by leafcutter bees, their nest cells “differ little in structure and nesting behavior,” a trait that makes the cells often difficult to identify. Yet the nest cell morphology of LACMRLP 388E provides one of the most important diagnostic tools to species-level. Nest cell construction of leafcutter bees is rarely closely examined or recorded. Where this has occurred [9], [10], [31], species-specific aspects of nest cell architecture have been documented. We surmise that there is indeed great morphological variation among *Megachile* species as has been demonstrated for another megachilid genus, *Osmia* [50]. A record of species-specific materials and nest structure may provide diagnostic tools useful in the field and for an organism that is often difficult to identify at an immature stage.

## Materials and Methods

### Excavation and Preparation

The cells, which were nested in chunky asphalt-impregnated matrix, were easier to excavate than if they had been preserved in more asphalt-concentrated matrix and therefore, were not exposed to solvent used in other preparation techniques. In addition, the immediate coating of the specimens with glyptol, an alkyd resin, upon discovery may have prevented further breakage during handling and preparation (Obermayr, Pit 91 field notes p. 1770, 1970).

### Micro-CT Scanning and Reconstruction

A 3D model was built for each pupae using Micro-CT data (Videos S1-6). Specimens were scanned on a SCANCO Micro-CT Scanner Model V1.2a. Linear Attenuation was 1/cm, 7.000000e+01 kVp with a slice thickness of 5 microns. Micro-CT slices were analyzed and 3D reconstructions of each bee built using Amira 5.4. LACMRLP 388Ea (male) was output as 2172 slices and reconstructed using 3,021,012 triangles (Videos S3, 5). LACMRLP 388Eb (female) was output as 2849 slices and reconstructed using 4,380,432 triangles (Videos S4, 6). To build 3D models of the nests, we scanned each nest on a MicroCAT II small animal CT system (Siemens Preclinical Solutions, Knoxville, TN, USA). Exposure settings were 70 kVp, 500 mAs, with a slice thickness of 2 mm and output as 436 slices. The female nest was built and output at 18,540 triangles. The male nest was built and output at 11,012 triangles. All models were cleaned and smoothed in Geomagic 10.

### Use of Comparative Material

The fossil specimens were also compared to material from the comprehensive collection at the USDA-ARS Pollinating Insect Research Lab. The plant materials used and specific architecture was examined from the nest cells of other *Megachile* species of similar, diminutive size that include western United States distribution.

### Estimation of Habitat Suitability at Present and During the LGM

#### Environmental space use

To investigate differences in environmental space use between *M. gentilis* and *M. onobrychidis*, we performed principal components analysis of bioclimatic variables, altitude, and Cartesian coordinates (*i.e.,* decimal degrees latitude and longitude) associated with distribution records. Contemporary environmental space use was estimated from recent specimen locality records (Dataset S1; National Pollinating Insect Database, Logan, UT) for eight informative bioclimatic variables: mean temperature of warmest quarter, mean temperature of coldest quarter, mean temperature of wettest quarter, mean temperature of driest quarter, precipitation of wettest quarter, precipitation of driest quarter, precipitation of warmest quarter, temperature annual range (Fig. S1; [51], http://worldclim.org). The contemporary (∼1,950–2,000 AD) bioclimatic variables were selected based on their ability to capture seasonal trends and species-specific differences between *M. gentilis* and *M. onobrychidis.* Differences in the distribution of the study species for each bioclimatic variable were tested with the Wilcoxon rank sum test with significance (*P*) based on an alpha of 0.95 and visualized with violin plots (Fig. S1). Data concentration ellipses were estimated using the k-means clustering method (*K* = 2) to visualize contemporary record assignment, as well as assign the environmental space of the La Brea Tar Pits during the LGM to either *M. gentilis* or *M. onobrychidis*.

#### Geographic distribution of habitat suitability

To estimate the geographic distribution of habitat suitability in the western U.S. at present and during the LGM (∼21,000 y BP), habitat suitability models (HSM) were constructed under the principle of maximum entropy with MaxEnt v3.3.1 [52]. Each species’ HSM was constructed from the specimen locality records and bioclimatic variables selected for the principal component and cluster analysis (see Environmental Space Use). Under the principle of niche conservatism [53], we estimated the geographic distribution of habitat suitability of both species during the LGM with analog bioclimatic variables of their contemporary bioclimatic niche. To provide a robust estimate of LGM habitat suitability, we allowed for the HSM to exceed contemporary bioclimatic extremes in the event that maximum entropy was achieved during model construction. Constructing LGM HSMs that are younger than the fossil records of the study species guarantees that both *M. gentilis* and *M. onobrychidis,* or a common *Litomegachile* ancestor were already in existence. The spatial resolution of all bioclimatic variables used in the models is 2.5 arc-minutes. Data processing and geographic visualization of the HSM were implemented in ArcGIS 10.1 (ESRI, Redlands, CA). Model performance was evaluated in MaxEnt using the area under curve (AUC) statistic and a jackknife of variable importance (Table S1). The AUC is constrained between zero and one where values closer to one suggest better model performance. Models were averaged over 50 replicates, with 10-fold cross validation for each replicate for model validation.

#### Statistical analysis

Outside of MaxEnt, all analyses were conducted in R v3.3.0 [54]. Principal components were visualized with the *scatterplot* function in the *car* library. The k-means clustering method was performed with the *kmeans* function in the *cluster* library. Violin plots were visualized with the *vioplot* library.

## Supporting Information

Figure S1 **Violin plots of eight bioclimatic variables associated with the distributions of** ***M. gentilis*** **and** ***M. onobrychidis*** **.** The width along each violin plot represents the frequency of specimen records associated with a measurement of the bioclimatic variable under observation. Wider widths reflect a higher frequency of specimen records, whereas thinner widths reflect a lower frequency of specimen records. Two-sample Wilcoxon tests were performed to test for precipitation/temperature differences between the species for each bioclimatic variable. Alpha levels for each test were set at alpha = 0.95. *P*<0.05 suggests significant differences in bioclimatic space use between the two species. M. gen = M. gentilis and M. ono = M. onobrychidis.
(TIFF)

Figure S2
**Contemporary habitat suitability distribution of** ***M. gentilis*** **and** ***M. onobrychidis*** **across an elevation gradient in southern California.** Kendall 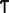 rank correlation coefficient estimates for *M. gentilis* revealed a positive and significant correlation between HS and Elevation (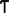 = 0.31, *P*<0.05), and a negative and non-significant correlation for *M. onobrychidis* (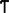 = 0.31, *P* = 0.52).(TIFF)

Table S1 **Analysis of bioclimatic variable importance for the MaxEnt habitat suitability models (HSM) of** ***M. gentilis*** **and** ***M. onobrychidis*** **.** Values represent averages over 50 replicate runs of each species’ habitat suitability model (HSM).
(PDF)

Dataset S1 **Natural history records of** ***M. gentilis*** **and** ***M. onobrychidis*** **utilized to construct environmental space use and habitat suitability models (HSM).** All specimens of each species present in the U.S. National Pollinating Insect Collection Database are presented in the table. *Megachile brevis onobrychidis* has been synonymized as *M. onobrychidis*. The former name is retained in the data table. Duplicate specimen records for each species per unique locality were removed for the final analyses.
(XLSX)

Video S1 **LACMRLP 388Ea. Rotating video nest of opaque to transparent nest cell containing male pupa.**
(MP4)

Video S2 **LACMRLP 388Eb. Rotating video nest of opaque to transparent nest cell containing female pupa.**
(MP4)

Video S3 **LACMRLP 388Ea. Rotating dorsal to ventral video of male pupa.**
(MP4)

Video S4 **LACMRLP 388Ea. Rotating dorsal to ventral video of female pupa.**
(MP4)

Video S5 **LACMRLP 388Ea. Rotating lateral video of male pupa.**
(MP4)

Video S6 **LACMRLP 388Eb Rotating lateral video of female pupa.**
(MP4)

Video S7 **LACMRLP 388Ea. Rotating quarter edge of nest cell.** This video shows cross-sections of leaves from different angles.
(MP4)

## References

[1] Latreille PA (1802–1803) Histoire naturelle, générale et particulière, des Crustacés et des Insectes, (Third edition). Paris: Dufart. 468 p.

[2] O’Toole C, Raw A (1991) Bees of the world. London: Blandford Publishing. 192 p.

[3] Munz PA, Keck DD (1959) A California flora. Berkeley and Los Angeles: University of California Press. 681 p.

[4] Baldwin BG, Goldman DH, Keil DJ, Patterson R, Thomas JR (2012) The Jepson manual. Vascular plants of California (Second edition). Berkeley, Los Angeles, and London: University of California Press. 1600 p.

[5] Michener CD (2007) The bees of the world (Second edition). Baltimore: The Johns Hopkins University Press, Baltimore. 953 p.

[6] Hurd PD, Michener CD (1955) The megachiline bees of California (Hymenoptera: Megachilidae). In: Linsley EG, Essig EO, Freeborn SB, Usinger RL editors. The Bulletin of the California insect survey (Volume 3). Berkeley and Los Angeles: University of California Press. 1–247 p.

[7] Krombein KV (1967) Trap-nesting wasps and bees: Life histories, nests, and associates (Volume 6). Washington, DC: Smithsonian Institution Press. 570 p.

[8] Michez D, Vanderplanck M, Engel M (2012) Fossil bees and their plant associates, In: Patiny S editor. Evolution of plant-pollinator relationships. Cambridge: Cambridge University Press. pp. 103–164.

[9] Kim JY (1992) Nest dimensions of two leaf-cutter bees (Hymenoptera: Megachilidae). Ann Entomol Soc Am 85: 85–90.

[10] Kim JY (2007) Disc size regulation in the brood cell building behavior of leaf-cutter bee, *Megachile tsurugensis* . Naturwissenschaften 94: 981–990. 17563863 10.1007/s00114-007-0277-4

[11] Engel MS, Perkovsky EE (2006) An Eocene bee in Rovno Amber (Hymenoptera: Megachilidae). Am Mus Novit 3506: 1–12.

[12] Sarzetti L, Labandeira CC, Genise JF (2008) A leafcutter bee tracefossil from the Middle Eocene of Patagonia Argentina and a review of megachilid (Hymenoptera) ichnology. Palaeontol 51: 933–941.

[13] Engel MS (1999) *Megachile glaesaria*, the first megachilid bee fossil from amber (Hymenoptera: Megachilidae). Am Mus Novit 3276: 1–13.

[14] Engel MS (2001) A monograph of the Baltic amber bees and evolution of the Apoidea (Hymenoptera). Bull Am Mus Nat Hist 259: 1–192.

[15] Engel MS, Grimaldi DA (2004) New light shed on the oldest insect. Nature 427: 627–630. 14961119 10.1038/nature02291

[16] Berry EW (1916) The Lower Eocene floras of southeastern North America. U.S.G.S. Prof Pap 91: 1–469.

[17] Berry EW (1931) An insect-cut leaf from the Lower Eocene. Am J Sci 21: 301–304.

[18] Brooks HK (1955) Healed wounds and galls on fossil leaves from the Wilcox deposit (Eocene) of western Tennessee. Psyche 62: 1–9.

[19] Lewis SE (1994) Evidence of leaf-cutting bee damage from the Republic sites (Middle Eocene) of Washington. J Paleontol 68: 172–173.

[20] Wappler T, Engel MS (2003) The middle Eocene bee faunas of Eckfeld and Messel, Germany (Hymenoptera: Apoidea). J Paleontol 77: 908–921.

[21] Cockerell TDA (1908) Fossil insect from Florissant, Colorado. Am Mus Nat Hist 24: 59–69.

[22] Wedmann S, Wappler T, Engel MS (2009) Direct and indirect fossil records of megachilid bees from the Paleogene of Central Europe (Hymenoptera: Megachilidae). Naturwissenschaften 96: 703–712. 19296064 10.1007/s00114-009-0525-x

[23] Litman JR, Danforth BN, Eardley CD, Praz CJ (2011) Why do leafcutter bees cut leaves? New insights into the early evolution of bees. Proc R Soc B 278: 3593–3600. 10.1098/rspb.2011.0365 PMC3189370 21490010

[24] Mitchell TB (1935) A revision of the genus *Megachile* in the Nearctic Region. Part II. Morphology of the male sternites and genital armature and the taxonomy of the subgenera *Litomegachile*, *Neomegachile* and *Cressoniella* (Hymenoptera: Megachilidae). Trans Am Entomol Soc (1890-) 61: 1–44.

[25] Bzdyk EL (2012) A revision of the *Megachile* subgenus *Litomegachile* Mitchell with an illustrated key and description of a new species (Hymenoptera, Meagchilidae, Megachilini). Zookeys 221: 31–61. 10.3897/zookeys.221.3234 PMC3487634 23129980

[26] Sheffield CS, Genaro JA (2013) A new species of *Megachile* (*Litomegachile*) from Cuba, the Antilles (Hymenoptera: Megachilidae). J Melittology 19: 1–17.

[27] Coope GR (2004) Several million years of stability among insect species because of, or in spite of, Ice Age climate instability? Philos Trans R Soc Lond B Biol Sci 359: 2009–214. 10.1098/rstb.2003.1393 PMC1693312 15101577

[28] Friscia AR, Van Valkenburgh B, Spencer L, Harris JM (2008) Chronology and spatial distribution of large mammal bones in Pit 91, Rancho La Brea. Palaios 23: 35–42.

[29] Cane JH, Gerdin SG, Wife G (1983) Mandibular gland secretions of solitary bees (Hymenoptera: Apoidea): Potential for nest cell disinfection. J Kan Entomol Soc 56: 199–204.

[30] Cane JH, Carlson RG (1984) Dufour’s gland triglycerides from *Anthophora*, *Emphoropsis* (Anthophoridae) and *Megachile* (Megachildae) bees (Hymentoptera: Apoidea). Comp Biochem Physiol 78B: 769–772. 10.1016/0305-0491(84)90132-9 6478803

[31] Strickler K, Scott VL, Fisher RL (1991) Comparative nesting ecology of two sympatric leafcutting bees that differ in body size (Hymenoptera: Megachilidae). J Kans Entomol Soc 69: 26–44.

[32] Michener CD (1953) The biology of a leafcutter bee (*Megachile brevis*) and its associates. Univ Kansas Sci Bull 35: 1659–1748.

[33] Packer L (1987) The triungulin larva of *Nemognatha (Pauronemognatha) punctulata* LeConte (Coleoptera: Meloidae) with a description of the nest of its host-*Megachile brevis pseudobrevis* Say (Hymenoptera: Megachilidae). J Kans Entomol Soc 60: 280–287.

[34] Ellis B, Daly DC, Hickey LJ, Johnson KR, Mitchell JD, et al.. (2009) Manual of leaf architecture. Ithaca: Comstock Publishing Associates. 216 p.

[35] Arnott HJ, Tucker SC (1963) Analysis of petal venation in Ranunculus. I. Anastomoses in *R. repens, V. pleniflorus* . Am J Bot 50: 821–830.

[36] Arnott HJ, Tucker SC (1964) Analysis of petal venation in Ranunculus. II. Number and position of dichotomies in *R. repens var. pleniflorus* . Bot Gaz 125: 13–26.

[37] Gustafsson MH (1955) Petal venation in the Asterales and related orders. Bot J Linn Soc 118: 1–18.

[38] Shaw CA (1982) Techniques used in excavation, preparation, and curation of fossils from Rancho La Brea. Curator 25: 63–77.

[39] Stock CS, Harris JM (1992) Rancho La Brea: A record of Pleistocene life in California. Los Angeles: Natural History Museum of Los Angeles County. 113 p.

[40] Warter JK (1979) The environment of Pit 91, Rancho La Brea, as interpreted by plant remains. Abs, Ann Mtg S Ca Acad Sci 87: 44.

[41] Shaw CS, Quinn JP (1986) Rancho La Brea: A look at coastal southern California’s past. Ca Geo 39: 123–133.

[42] Robert C (2004) Later Quaternary variability of precipitation in southern California and climatic implications: clay mineral evidence from the Santa Barbara Basin, ODP Site 893. Quaternary Sci Rev 23: 1029–1040.

[43] Bischoff JL, Cummins K (2001) Wisconsin glaciation of the Sierra Nevada (79,000–15,000 yr BP) as recorded by rock flour in sediments of Owens Lake, California. Quaternary Res 55: 14–24.

[44] Miller SE, Gordon RD, Howden HF (1981) Reevaluation of Pleistocene scarab beetles from Rancho La Brea, California (Coleoptera: Scarabaeidae). Proc Entomol Soc Wash 83: 625–630.

[45] Holden AR, Harris JM, Timm RM (2013) Paleoecological and taphonomic implications of insect-damaged Pleistocene vertebrate remains from Rancho La Brea, southern California. PloS one 8(7): e67119. 23843988 10.1371/journal.pone.0067119 PMC3700975

[46] Heusser L (1998) Direct correlation of millennial-scale changes in western North American vegetation and climate with changes in the California current system over the past ∼60 kyr. Paleoceanogr 13: 252–262.

[47] Coltrain JB, Harris JM, Cerling TE, Ehleringer JR, Dearing MD, et al (2004) Rancho La Brea stable isotope biogeochemistry and its implications for the palaeoecology of Late Pleistocene, coastal southern California. Palaeogeogr, Palaeoclimatol, Palaeoecol 205: 199–219.

[48] Jacobs DK, Haney TA, Louie KD (2004) Genes, diversity, and geologic process on the Pacific Coast. Annu Rev Earth Planet Sci 32: 602–652.

[49] Doyen JT, Miller SE (1980) Review of Pleistocene darkling ground beetles of the California asphalt deposits (Coleoptera: Tenebrionidae, Zopheridae). Pan-Pac Entomol 56: 1–10.

[50] Cane JH, Griswold T, Parker FD (2007) Substrates and materials used for nesting by North American *Osmia* bees (Hymenoptera: Apiformes: Megachilidae). Ann Entomol Soc Am 100: 350–358.

[51] Hijmans RJ, Cameron SE, Parra JL, Jones PG, Jarvis A (2005) Very high resolution interpolated climate surfaces for global land areas. Int J Climatol 25: 1965–1978.

[52] Phillips SJ, Anderson RP, Schapire RE (2006) Maximum entropy modeling of species geographic distributions. Ecol Model 190: 231–259.

[53] Peterson AT, Soberón J, Sánchez-Cordero V (1999) Conservatism of ecological niches in evolutionary time. Science 285: 1265–1267. 10455053 10.1126/science.285.5431.1265

[54] R Core Team (2012) R A language and environment for statistical computing. R Foundation for Statistical Computing, Vienna, Austria. ISBN 3-900051-07-0. Available: http://www.R-project.org.

