# Supplementary figures and images for: Leafcutter Bee Nests and Pupae from the Rancho La Brea Tar Pits of Southern California: Implications for Understanding the Paleoenvironment of the Late Pleistocene
Source: PLoS One. 2014 Apr 9;9(4):e94724. doi: 10.1371/journal.pone.0094724 (PMC3981822; doi:10.1371/journal.pone.0094724)

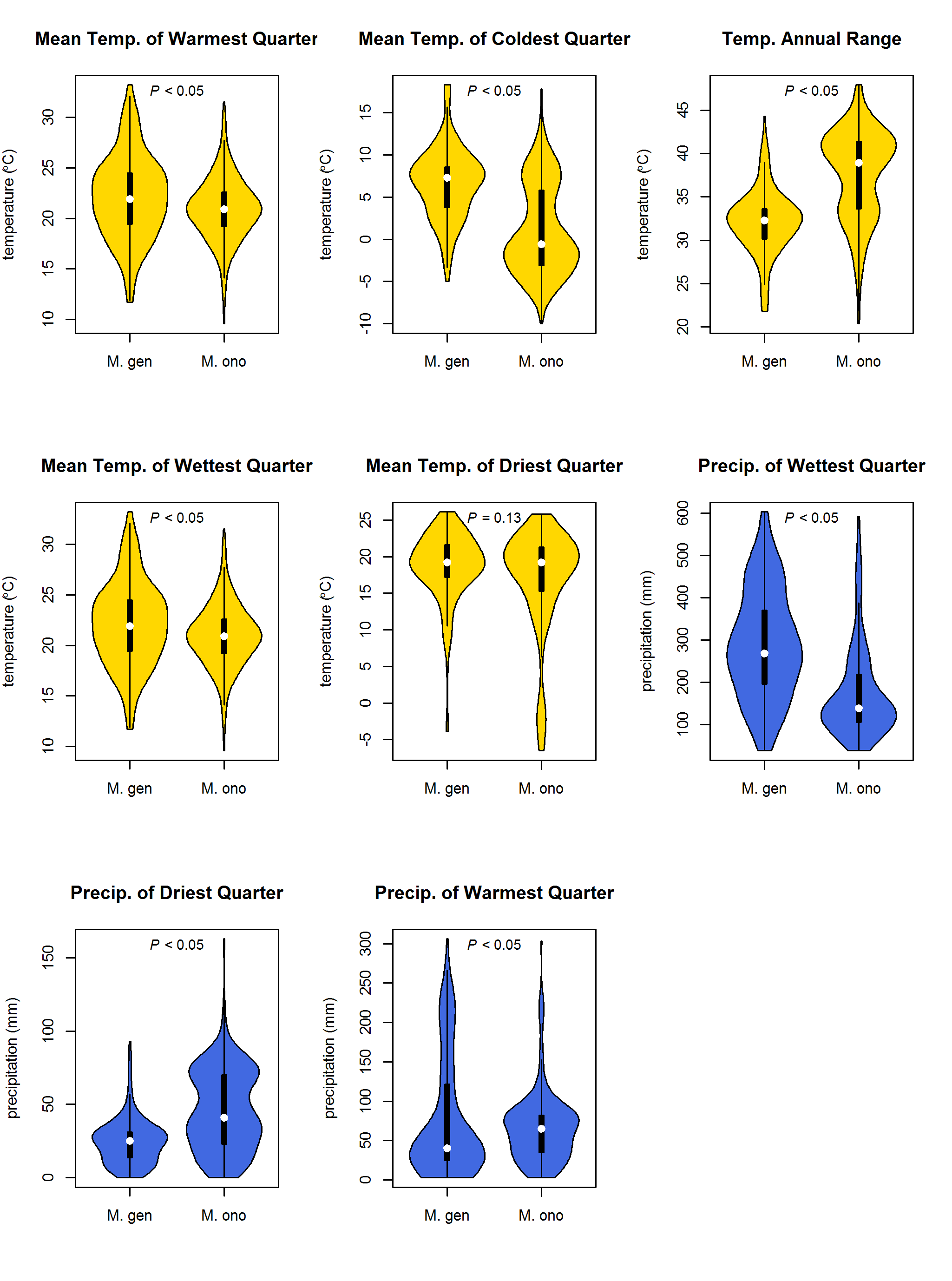

Supplement: Figure S1 — Violin plots of eight bioclimatic variables associated with the distributions of M. gentilis and M. onobrychidis . The width along each violin plot represents the frequency of specimen records associated with a measurement of the bioclimatic variable under observation. Wider widths reflect a higher frequency of specimen records, whereas thinner widths reflect a lower frequency of specimen records. Two-sample Wilcoxon tests were performed to test for precipitation/temperature differences between the species for each bioclimatic variable. Alpha levels for each test were set at alpha = 0.95. P<0.05 suggests significant differences in bioclimatic space use between the two species. M. gen = M. gentilis and M. ono = M. onobrychidis. (TIFF) [file pone.0094724.s001.tiff]

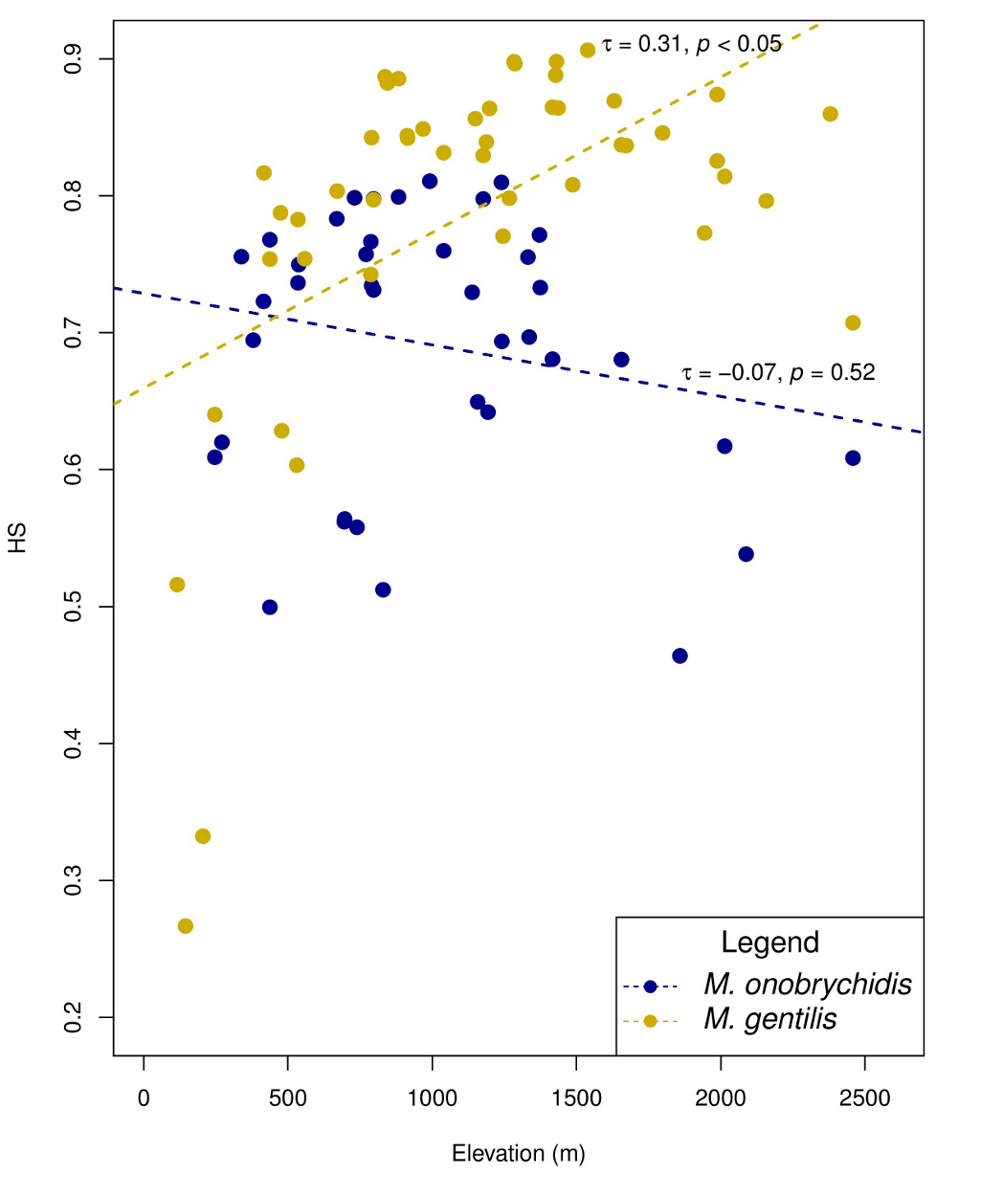

Supplement: Figure S2 — Contemporary habitat suitability distribution of M. gentilis and M. onobrychidis across an elevation gradient in southern California. Kendall rank correlation coefficient estimates for M. gentilis revealed a positive and significant correlation between HS and Elevation ( = 0.31, P<0.05), and a negative and non-significant correlation for M. onobrychidis ( = 0.31, P = 0.52). (TIFF) [file pone.0094724.s002.tiff]
