# Supplementary material for: Leafcutter Bee Nests and Pupae from the Rancho La Brea Tar Pits of Southern California: Implications for Understanding the Paleoenvironment of the Late Pleistocene
Source: PLoS One. 2014 Apr 9;9(4):e94724. doi: 10.1371/journal.pone.0094724 (PMC3981822; doi:10.1371/journal.pone.0094724)
Supplement: Table S1 — Analysis of bioclimatic variable importance for the MaxEnt habitat suitability models (HSM) of M. gentilis and M. onobrychidis . Values represent averages over 50 replicate runs of each species’ habitat suitability model (HSM). (PDF) [file pone.0094724.s003.pdf]

Supporting Information Table 1. Analysis of bioclimatic variable importance for the habitat suitability models of *M. gentilis* and *M. onobrychidis*.

		**Jackknife analysis**			
	**% contribution**	**Train gain with[1]**	**Train gain without[2]**	**Test gain with[3]**	**Test gain without[4]**
M. gentilis Habitat Suitability Model					
Mean Temperature of Warmest Quarter	0.86	2.07	0.05	2.12	0.03
Mean Temperature of Coldest Quarter	11.04	2.01	0.67	2.04	0.70
Precipitation of Wettest Quarter	6.99	2.00	0.04	1.99	0.02
Precipitation of Driest Quarter	21.74	1.94	0.81	2.14	0.56
Precipitation of Warmest Quarter	31.76	2.03	1.06	2.08	1.06
Temperature Annual Range*	16.18	2.02	0.71	2.08	0.76
Mean Temperature of Wettest Quarter	2.44	2.07	0.61	2.13	0.68
Mean Temperature of Driest Quarter	8.99	2.00	0.67	2.04	0.68
M. onobrychidids Habitat Suitability Model					
Mean Temperature of Warmest Quarter	1.4787	1.7403	0.1674	2.0587	0.143
Mean Temperature of Coldest Quarter	3.9816	1.7378	0.3641	2.0722	0.3622
Precipitation of Wettest Quarter	4.5882	1.6758	0.4851	1.969	0.511
Precipitation of Driest Quarter	2.3827	1.7315	0.4892	2.0437	0.5273
Precipitation of Warmest Quarter	67.8178	1.6965	1.3479	1.987	1.3861
Temperature Annual Range*	2.9892	1.7181	0.2196	2.0194	0.2747
Mean Temperature of Wettest Quarter	5.2406	1.7035	0.7823	2.0125	0.8024
Mean Temperature of Driest Quarter	11.5212	1.6723	0.5301	1.9397	0.5184

*Max Temperature of Warmest Month – Min Temperature of Coldest Month.

[1]Training gain achieved with each climate variable in isolation.

[2]Training gain achieved excluding each variable in turn.

[3]Test gain achieved with each climate variable in isolation.

[4]Test gain achieved excluding each variable in turn.
